# The Neurobehavioral Impact of Zinc Chloride Exposure in Zebrafish: Evaluating Cognitive Deficits and Probiotic Modulation

**DOI:** 10.3390/toxics13030193

**Published:** 2025-03-08

**Authors:** Madalina Ene, Alexandra Savuca, Alin-Stelian Ciobica, Roxana Jijie, Irina Luciana Gurzu, Luminita Diana Hritcu, Ionut-Alexandru Chelaru, Gabriel-Ionut Plavan, Mircea Nicusor Nicoara, Bogdan Gurzu

**Affiliations:** 1 Department of Biology, Faculty of Biology, “Alexandru Ioan Cuza” University of Iasi, Carol I Avenue, 20A, 700505 Iasi, Romania; ene.emadalina@yahoo.ro (M.E.); alin.ciobica@uaic.ro (A.-S.C.); gabriel.plavan@uaic.ro (G.-I.P.); mirmag@uaic.ro (M.N.N.); 2 Doctoral School of Geosciences, Faculty of Geography and Geology, “Alexandru Ioan Cuza” University of Iasi, Carol I Avenue, 20A, 700505 Iasi, Romania; chelaru.alexandru@yahoo.com; 3 Doctoral School of Biology, Faculty of Biology, “Alexandru Ioan Cuza” University of Iasi, Carol I Avenue, 20A, 700505 Iasi, Romania; 4 Academy of Romanian Scientists, Splaiul Independentei Avenue No. 54, Sector 5, 050094 Bucharest, Romania; 5 Center of Biomedical Research, Romanian Academy, Carol I Avenue, No. 8, 700505 Iasi, Romania; 6 “Ion Haulica” Institute, Apollonia University, Păcurari Street 11, 700511 Iasi, Romania; 7 Research Center on Advanced Materials and Technologies, Department of Exact and Natural Sciences, Institute of Interdisciplinary Research, “Alexandru Ioan Cuza” University of Iasi, 700506 Iasi, Romania; roxanajijie@yahoo.com; 8 Department of Preventive Medicine and Interdisciplinarity, Faculty of Medicine, University of Medicine and Pharmacy “Grigore T. Popa”, 16th Universitatii Street, 700115 Iasi, Romania; irina-luciana.gurzu@umfiasi.ro; 9 Internal Medicine Clinic, “Ion Ionescu de la Brad” University of Life Sciences, Sadoveanu Alley No. 3, 700490 Iasi, Romania; lumidih@yahoo.com; 10 Department of Morfofunctional Sciences, Faculty of Medicine, “Grigore T. Popa” University of Medicine and Pharmacy, 16th Universitatii Street, 700115 Iasi, Romania; bgurzu@yahoo.com

**Keywords:** zinc chloride, probiotics, toxicity, zebrafish, behavior

## Abstract

Zinc contamination in aquatic environments has become a growing concern due to its potential to bioaccumulate and induce neurotoxic effects in aquatic organisms. As an essential trace element, zinc plays a crucial role in various physiological processes, but excessive exposure can disrupt the gut–brain axis, leading to cognitive and behavioral impairments. Recent studies have suggested that probiotics may offer protective effects against environmental neurotoxins by modulating the gut microbiota and associated neurological functions. The zebrafish (*Danio rerio*) has emerged as a valuable model organism for studying the biological mechanisms underlying neurotoxicity and potential therapeutic interventions. This study aimed to assess the effects of probiotics on cognitive impairments induced by zinc chloride (ZnCl_2_) exposure in zebrafish. Specifically, zebrafish were exposed to ZnCl_2_ at concentrations of 0.5 mg/L and 1.0 mg/L for 96 h, followed by a 7-day post-exposure period to probiotics (*Bifidobacterium longum*, *Bifidobacterium animalis lactis*, *Lactobacillus rhamnosus*). ZnCl_2_ exposure at these concentrations is already known to induce behavioral and neuromotor deficits resembling Alzheimer’s disease-like symptoms in zebrafish models, making it a suitable model for evaluating the neuroprotective potential of probiotics. Behavioral assessments including sociability tests along with short- and long-term memory evaluations were conducted using EthoVision XT 16 software. Memory tests demonstrated that ZnCl_2_ exposure impaired cognitive functions, while probiotic treatment did not significantly ameliorate these deficits. In the social behavior test, ZnCl_2_ at 0.5 mg/L resulted in a marked decrease in sociability, whereas exposure to 1.0 mg/L did not induce significant changes. However, post-exposure probiotic administration following ZnCl_2_ intoxication at 1.0 mg/L exhibited an anxiolytic effect on zebrafish. These findings suggest that probiotics may exhibit partial neurobehavioral benefits following zinc chloride-induced toxicity, particularly in mitigating anxiety-like behaviors rather than cognitive deficits. Further investigations are needed to elucidate the precise mechanisms by which probiotics interact with the gut–brain axis in the context of heavy metal neurotoxicity.

## 1. Introduction

The contamination of soil [1] and water resources [2] with zinc (Zn), as well as other heavy metals, exacerbated by the impacts of climate change [3], represents a growing environmental issue; this contamination can arise from both natural processes and anthropogenic activities [4]. Excessive Zn concentrations, despite Zn’s essential role for organisms, disrupt homeostasis and negatively affect ecosystems and human health [5].

Elevated Zn levels can result from contaminated water near industrial sites, Zn-coated pipes, excessive Zn supplements, or occupational exposure to Zn dust or fumes in industrial settings [6,7]. Zinc chloride (ZnCl_2_) is a versatile and widely used chemical in soldering, fireproofing, wood preservation, medicine, textile processing, agriculture, water treatment, and manufacturing products like batteries, cement, and golf balls [8,9]. It also serves as an herbicide, antiseptic, textile mordant, chemical intermediate, and a key component in military and firefighting smoke bombs [8,10].

Published data underline both the useful and noxious effect of Zn on nervous system activity [4,10]. Exposure to zinc induces significant toxicity in zebrafish, impacting various developmental stages. Embryonic and larval exposure studies revealed a significant increase in mortality at Zn^2+^ concentrations of 2 mg/L and higher after 168 h (*p* < 0.05). Furthermore, zinc exposure induced a range of larval malformations, including tail curving, spinal curvature, and pericardial edema, with significant increases in malformation rates observed at concentrations of 1, 2, 4, and 8 mg/L (*p* < 0.05) [11]. Acute toxicity tests on adult zebrafish demonstrated a concentration-dependent increase in mortality. While no mortality was observed in controls, 100% mortality occurred within 24 h at 15 mg/L ZnCl_2_. The 96 h medial lethal concentration for ZnCl_2_ was estimated to be approximately 6 mg/L (ppm) [12]. Post-hatching lethality also occurred in a concentration-dependent manner, with an LC50 of 2.31 mg/L (95% CI: 1.81–3.05) and a lowest observed effect concentration of 1.5 mg/L. Growth inhibition was observed at 1.5 mg/L at both 15- and 30-days post-hatching [13].

Zinc accumulation significantly alters the zebrafish’s gut microbiota in two key ways: increasing the overall microbial abundance while simultaneously reducing diversity. Specifically, zinc exposure leads to a decline in beneficial bacteria, a rise in opportunistic pathogens, and an increase in microorganisms associated with oxidative stress [14]. Specific strains of bacteria, including *Bifidobacterium longum*, *Bifidobacterium animalis lactis*, and *Lactobacillus rhamnosus*, have demonstrated potential in interacting with the gut microbiota and influencing its composition and function [15]. The mechanisms by which these probiotic strains exert their beneficial effects are multifaceted. They can compete with heavy metals for binding sites in the gut, reducing their absorption into the body [16]. Some strains can even bind directly to heavy metals, facilitating their excretion. Furthermore, these probiotics can produce short-chain fatty acids (SCFAs), which have been shown to have neuroprotective effects and can influence brain function through the gut–brain axis. They may also modulate the immune system, reducing the inflammation and oxidative stress induced by heavy metal exposure [17]. For example, *Lactobacillus rhamnosus* has been studied for its ability to bind to certain heavy metals, while *Bifidobacterium longum* has shown promise in reducing inflammation [18]. *Bifidobacterium animalis lactis* has demonstrated positive effects on gut barrier function [19].

Ensuring balanced zinc levels regulates oxidative stress and neuroinflammatory responses [20]. Recent findings suggest that dysregulation of Zn^2+^ homeostasis contributes to the pathogenesis of various central nervous system disorders, such as Alzheimer’s disease, Parkinson’s disease, and multiple sclerosis, as well as psychiatric conditions like depression and schizophrenia and neurological disorders including epilepsy [21]. Exposure to heavy metals can interfere with the gut–brain axis [22], a bidirectional communication system linking the gut microbiota with the CNS [23]. This disruption has been linked to cognitive and behavioral impairments [24,25].

Probiotics, beneficial microorganisms that contribute to gut health [26], are being increasingly explored for their ability to influence communication between the gut and the CNS [27]. By reducing inflammation and oxidative stress, probiotics may indirectly counteract the neurotoxic effects of metals [28]. However, the extent of probiotics’ protective effects, particularly against zinc neurotoxicity, remains unclear.

Recent studies using zebrafish (*Danio rerio*) have examined the influence of gut microbiota on neurodevelopment, behavior, and disease susceptibility, highlighting this experimental model’s efficacy for exploring microbial contributions to gut–brain interaction [24,29,30]. Zebrafish are a well-known model organism for neurobehavioral and toxicological research on large populations with controlled environmental exposures and can be used following the “3Rs” principles [29,31,32].

Taking into consideration published data, a zebrafish experimental model was used to evaluate the effects of zinc neurotoxicity and potential probiotics’ beneficial effects. Behavioral assessments, including tests for sociability, short-term memory, and long-term memory, were conducted on zebrafish adults exposed to zinc chloride, both with and without probiotics.

## 2. Materials and Methods

### 2.1. Ethical Note

Animals were treated and maintained in accordance with the EU Commission Recommendation (2007), Directive 2010/63/EU of the European Parliament, and Council guidelines of 22 September 2010 on the accommodation, care, and protection of animals used for experimental and other scientific purposes. The protocol we followed received approval from the Ethics Commission of the Faculty of Biology, “Alexandru Ioan Cuza” University, Iasi, No. 928/05.04.2023, which was authorized as a unit for animals used for scientific purposes (with No. 4/03.06.2020) by the Regional Branch of the National Veterinary Sanitary and Food Control Agency.

### 2.2. Animal Maintenance

A total of 60 wild-type zebrafish adults (6 months old) of both sexes (50/50 sex ratio) were acquired from a local authorized fish farm (Iasi, Romania). The zebrafish were acclimatized in groups of 15 for two weeks in 10 L tanks containing dechlorinated water, which was changed daily. Fish were fed twice per day with commercial flakes for fish (≈2–3 mm diameter) (TetraMin Flakes, Tetra, Germany). The water conditions were as follows: temperature 26 ± 1 °C, pH 7.5, dissolved oxygen 7.20 mg/L, ammonia concentration < 0.004 ppm, and conductivity 500 μS. The light–dark exposure cycle was set to 12: 12 h. The tank water was filtered continuously to avoid accumulation of organic toxins and aerated with oxygen pumps.

### 2.3. Chemical Compounds Administration

Zinc chloride (ZnCl_2_, Chimreactiv Romania, min. 99% purity) was administered by dissolving it in the fish’s environment. The concentrations of zinc chloride used were 0.5 mg/L and 1.0 mg/L, respectively. These concentrations were chosen based on findings in the literature. To ensure that the dose of interest was consistently present in the fish water, the zinc chloride solution was prepared and administered daily de novo.

Probiotics (a pharmaceutical-based product containing *Bifidobacterium animalis lactis, Bifidobacterium longum*, and *Lactobacillus rhamnosus* strains—1 × 10 CFU, fructooligosaccharides—300 mg, and vitamin C—12 mg per sachet) were administered by dissolving them in the fish’s environment. To avoid any conflicts of interest for these products, the commercial label remains anonymous. For the probiotic exposure, the solution was prepared de novo daily to ensure that the dose of interest was obtained by dissolving one sachet in 10 L of water (10L representing the entire fish environment).

### 2.4. Experimental Design

**Figure 1:**
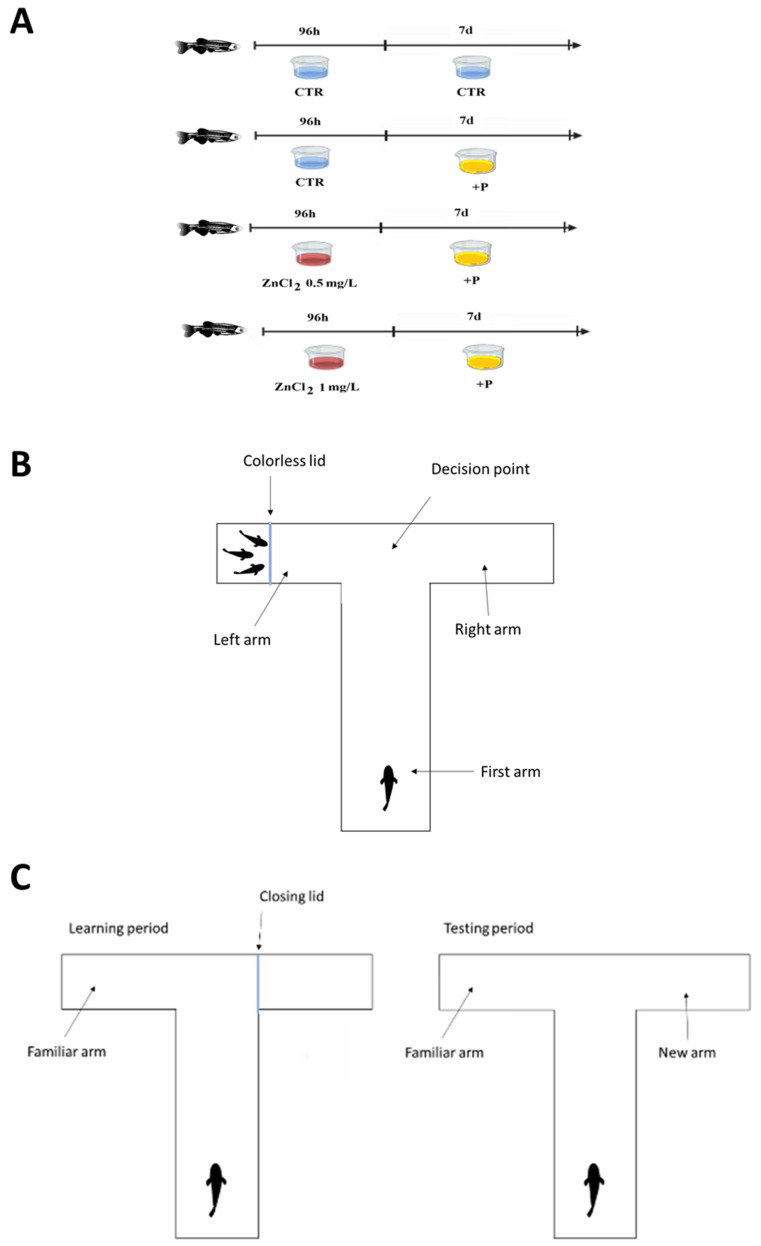
The visual representation of the experimental design: (**A**) described experimental design, timeline, and groups. (CTR, control; ZnCl_2_, zinc chloride; P, probiotics; h, hours; d, days); (**B**) the initial experimental setup representation for social preference test; (**C**) the experimental setup representation for short-term memory test.

Adult zebrafish were randomly assigned to 4 experimental groups (*n* = 15/group) as follows: CTR (control group), 0.5 mg/L ZnCl_2_ + P (0.5 mg/L ZnCl_2_ exposure for 96 h, followed by exposure to probiotics for 7 days), 1.0 mg/L ZnCl_2_ + P (1.0 mg/L ZnCl_2_ exposure for 96 h, followed by exposure to probiotics for 7 days), P (probiotics exposure for 7 days) (Figure 1A). The effects of 0.5 mg/L and 1.0 mg/L ZnCl_2_ concentrations and subsequently the effects of probiotics on zinc exposure were assessed, as well as the effects of probiotics versus control group.

#### 2.4.1. Social Preference Test

The social preference test was conducted using a cross-maze labyrinth, which was modified into a “T” maze by sealing one arm with a colorless plastic slit. The maze consisted of two arms (left and right) and a starting arm where the start box was placed (Figure 1B). To assess social behavior, three zebrafish were placed in a box located at the left arm of the maze, with the box constructed using a colorless plastic slit. The duration of the test was 5 min per individual.

#### 2.4.2. Short-Term Memory Test

The memory test was conducted using the same cross-maze labyrinth, modified into a “T” maze by sealing one arm. The maze consisted of three arms: a left arm, a right arm, and a main arm, where the start box was placed. The right arm was closed with a colorless slit so that the subject had access only to the main arm and the left arm. The subject was placed in this maze for 4 min.

After one hour, the slit was removed so that the maze would be in the shape of the letter “T” and the subject was placed in the maze for analysis of the amount of time that was spent in the right (new) arm and exploration, monitored with EthoVision XT 16 software (Noldus, Wageningen, The Nederland). The maze thus offers a direct choice to the subject to enter the previously accessible arm (the left one) or to enter the newly accessible one (the right one). The test reveals the subject’s learning and memory capacity. The experimental setup is represented in Figure 1C.

#### 2.4.3. Long-Term Memory Test

The long-term memory test was conducted in a 10 L rectangular tank (30 × 30 × 30 cm) in which a silver cube-shaped object was initially placed as a familiar object. The subject was placed in the tank for 5 min each day for 7 days to ensure learning. Subsequently, a new object, a black sphere, was added to the tank. The subject was returned to the tank with the two objects for a 5 min period, during which its behavior was monitored using EthoVision XT 16 software (Noldus, Wageningen, The Nederland). Exploration behavior, along with the time spent near the familiar and new objects, was used to assess the fish’s memory capacity.

#### 2.4.4. Statistical Analysis

The normality and distribution of the data were assessed using the Shapiro–Wilk test with GraphPad Prism 9 software (San Diego, CA, USA). Multiple group comparisons were performed using one-way ANOVA followed by Tukey’s test. Data are presented as the mean ± SEM, with a *p*-value of <0.05 considered statistically significant.

## 3. Results

### 3.1. Social Preference Test

This test examined the distance moved, velocity, inactivity time, number of entries into the conspecific arm of the maze (represented by the left arm), and the cumulative time spent in all arms of the maze and at the decision point for the fish. The test was performed in all four groups after ZnCl_2_ administration at 0.5 mg/L and 1.0 mg/L, respectively, and after probiotic administration in assigned groups.

**Figure 2:**
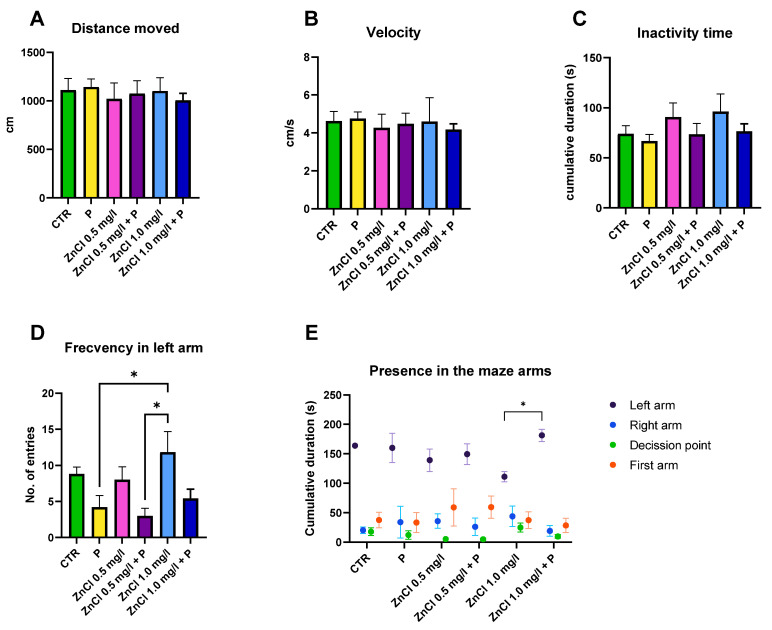
Graphical representation of behavioral trends observed in response to zinc chloride and probiotic interventions during the social preference test. (**A**) Distance moved; (**B**) Velocity; (**C**) Inactivity time; (**D**) Number of entries in the conspecific arm (left arm); (**E**) Time spent in the maze arms. Data are presented as mean ± SEM, with statistical significance set at *p* < 0.05 (*).

The distance moved and velocity of each group presented no remarkable changes compared to one another after the administration of ZnCl_2_, nor after the probiotics (Figure 2A,B).

The inactivity time registered by the two groups that received ZnCl_2_ is slightly elevated in comparison to the control and probiotics groups, though not statistically significant. After the probiotic treatment, the ZnCl_2_-exposed groups show a decrease in the duration of inactivity time. Probiotics alone did not affect this parameter (Figure 2C).

The number of entries in the left arm (with the conspecifics) was discrepant among the groups. There was a statistically significant difference (*p* = 0.048) between the probiotics group and the ZnCl_2_ 1.0 mg/L group. Another significant difference was observed between the ZnCl_2_ 1.0 mg/L group and the ZnCl_2_ 0.5 mg/L + P group (*p* = 0.016). Moreover, it can be observed that no changes occurred in this parameter in the case of exposure to ZnCl_2_ 0.5 mg/L (Figure 2D).

The presence of fish in the maze arms was analyzed using a two-way ANOVA comparison test, which showed that the time spent in the left arm increased significantly in the case of exposure to ZnCl_2_ 1.0 mg/L after the administration of probiotics (*p* = 0.018) (Figure 2E).

Other significant differences for the social preference test within the test groups were between the control group and the one to which only the probiotics were administered in the presence in the left arm and all other arms (*p* < 0.001); slight differences occur in the case of ZnCl_2_ administration where, for the ZnCl_2_ 0.5 mg/L group, a significant difference was highlighted between the time spent in the left arm vs. right arm/decision point (*p* < 0.001) and also between the left arm vs. first arm (*p* = 0.006), thus denoting the fact that the fish spent more time in the starting arm than they did in the control and P groups. The *p* value in this situation decreased slightly to *p* = 0.002 after probiotic administration. In the case of the ZnCl_2_ 1.0 mg/L group, the significant differences are weaker, so that for left arm vs. right arm (*p* = 0.032), left arm vs. decision point (*p* = 0.003), and left arm vs. first arm (*p* = 0.014), after the administration of the probiotics to this group, the *p* values of the same comparisons reached *p* < 0.001.

### 3.2. Short-Term Memory Test

The key parameters measured during this test included velocity, total distance traveled, and the duration of time spent in each arm of the maze. The test was performed in all for groups after administration of 0.5 mg/L and 1.0 mg/L ZnCl_2_, respectively, in the appropriate groups and after probiotic administration in the assigned groups.

**Figure 3:**
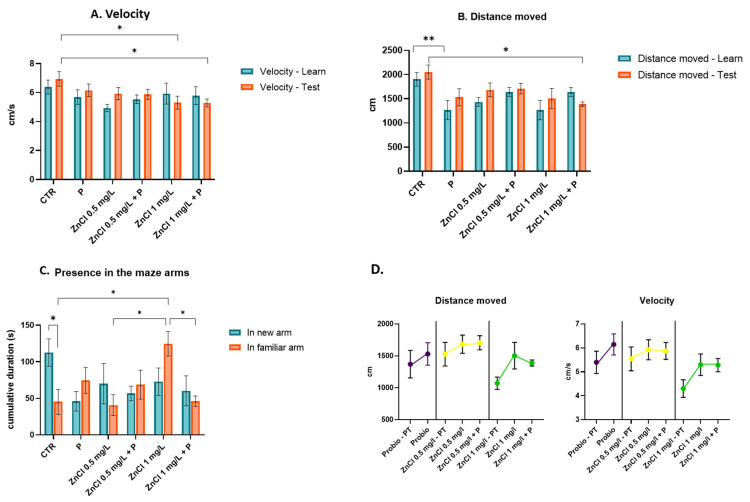
Graphical representation of behavioral patterns in response to zinc chloride and probiotic treatments during the short-term memory test: velocity (**A**); distance moved (**B**); presence in the maze arms (**C**); baseline characteristics of zebrafish swimming behavior within the treatments (**D**). PT = Pretreatment. Data are expressed as mean ± SEM and a significance level of *p* < 0.05 was considered statistically significant (*) and *p* < 0.01 = **.

The velocity in the learning stage of the test did not show any significant differences between groups. During the test phase, statistically significant differences were observed between the control group and the ZnCl_2_ 1.0 mg/L group (*p* = 0.039), as well as between the control group and the ZnCl_2_ 0.5 mg/L + P group (*p* = 0.037, Figure 3A).

The distance moved significantly decreased between the control and probiotics groups during the learning phase (*p* = 0.01, Figure 3B). During the test phase, a significant decrease was registered in the ZnCl_2_ 1 mg + P group in comparison to the control group (*p* = 0.017).

The presence in the maze arms revealed no significant differences between the time spent in the familiar arm vs. the new arm except in the control group (*p* = 0.028). The presence in the familiar arm was statistically significant between groups as follows: control group vs. ZnCl_2_ 1.0 mg/L group (*p* = 0.019), ZnCl_2_ 0.5 mg/L group vs. ZnCl_2_ 1.0 mg/L group (*p* = 0.012), and ZnCl_2_ 1.0 mg/L vs. ZnCl_2_ 0.5 mg/L + P group (*p* = 0.021, Figure 3C).

The baseline characteristics of zebrafish swimming behavior (distance moved and velocity) in the testing period within the treatments are presented in Figure 3D.

### 3.3. Long-Term Memory Test

The long-term memory test was performed in all four groups after ZnCl_2_ administration in the ZnCl_2_ 0.5 mg/L and ZnCl_2_ 1.0 mg/L groups, respectively, and after probiotic administration in the ZnCl_2_ 0.5 mg/L, ZnCl_2_ 1.0 mg/L, and P groups.

**Figure 4:**
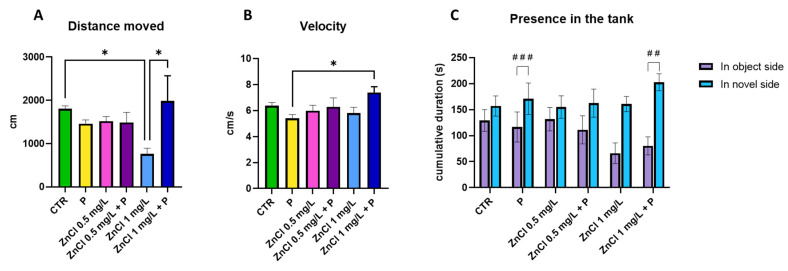
Graphical representation of behavioral patterns in response to zinc chloride and probiotic treatments during the long-term memory test. (**A**) Distance moved; (**B**) Velocity; (**C**) Time spent in the sides of the tank (object side vs. novel side). Data are presented as mean ± SEM, with significance levels denoted as follows: *, *p* < 0.05; ##, *p* = 0.002; ###, *p* < 0.001.

The distance moved showed significantly lower values between the control group and ZnCl_2_ 1.0 mg/L group (*p* = 0.046) and a significant increase after the administration of probiotics between the ZnCl_2_ 1.0 mg/L group and ZnCl_2_ 1.1 mg/L + P group (*p* = 0.018, Figure 4A). Significant increases in velocity were noted in the ZnCl_2_ 1.0 mg/L + P group compared with the probiotics (P) group (*p* = 0.044, Figure 4B).

The behavior of the groups was notably different when there was a familiar object (the silver cube) and a new object (the black sphere) in the tank. A statistically significant difference was observed in the time spent near the familiar object (object side) and near the new object (novel side) in the probiotics (P) group (*p* < 0.001) and in the ZnCl_2_ 1.0 mg/L + P group (*p* = 0.002, Figure 4C).

**Figure 5:**
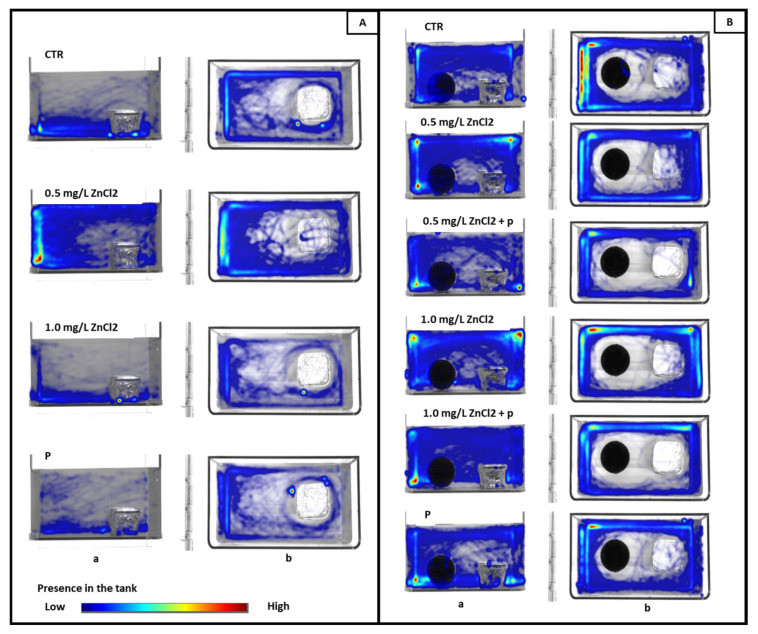
Heatmaps visually representing the time spent and activity patterns near the familiar object in the learning period (the silver cube-shaped object) (**A**) and the new object in the testing period (the black sphere) (**B**). CTR, the control group; P, the probiotic group; 0.5 mg/L ZnCl_2_ and 1 mg/L ZnCl_2_, zinc-treated groups; 0.5 mg/L ZnCl_2_ + P and 1 mg/L ZnCl_2_ + P, probiotic treatment after initial zinc chloride administration. (a—lateral view and b—top view). Where no color variation is present in the tank, the presence of fish was not detected in those locations.

For a better understanding and visualization of the effects of both zinc exposure and probiotic administration in the long-term memory test, Figure 5 shows representative heatmaps for each group during the test; the heatmaps were generated using EthoVision XT software. Figure 5A displays heatmaps for the learning period, showing the spatial distribution of activity in the presence of the familiar object for each experimental group, including the control group (CTR), zinc-treated groups (ZnCl_2_ 0.5 mg/L and ZnCl_2_ 1 mg/L), and the probiotic group (P). Figure 5B shows heatmaps where a black sphere (new object) was added to the tank. These heatmaps reflect differences in exploration and activity patterns across the same groups as in 5A. Figure 5B presents heatmaps of the novel object test performed immediately after zinc administration to the 0.5 mg/L ZnCl_2_ and 1.0 mg/L ZnCl_2_ groups, as well as after probiotic administration to the 0.5 mg/L ZnCl_2_, 1.0 mg/L ZnCl_2_, and P (probiotics) groups. The time spent and activity patterns of ZnCl_2_-exposed zebrafish before and after administration of probiotics (+P) are different than those of the control group (CTR) and the probiotic group (P), emphasizing the impact of probiotics on spatial activity and memory-related behavior.

## 4. Discussion

The term “bio-toxic effects of heavy metals” describes the detrimental consequences these elements have on human health when consumed in amounts that surpass biologically established safety limits [33]. Heavy metals have been repeatedly linked with neurodegenerative diseases over the years [34]. Zinc chloride has a wide range of applications, particularly in the food industry, where studies indicate that the addition of zinc chloride to packaged natural table olives can promote consumption by reducing desirable sensory characteristics, resulting in healthier products [35]. In addition, recent studies show that the use of ZnCl_2_ can enhance the antioxidant and vitamin content of peanut seeds [36], making human exposure to this substance much more likely through dietary intake. Furthermore, zinc chloride is generally recognized as safe in food [37]. However, previous studies have shown that exposure to certain low concentrations of zinc chloride may induce Alzheimer’s-like symptoms in adult zebrafish [12]. Also, the neurotoxicity mechanism of ZnCl_2_ exposure was previously demonstrated by Sarasamma et al. Their findings suggest that even at low concentrations, zinc induces decreases in phosphorylated Tau (p-Tau) and amyloid beta (amyloid β) protein levels, proteins that are also used as biomarkers in Alzheimer’s disease. Moreover, a significant inhibition of the neurotransmitter acetylcholine (ACh) was observed; this neurotransmitter is associated with various physiological and behavioral processes (e.g., motor activity, memory) via activating metabotropic muscarinic and ionotropic nicotinic cholinergic receptors, along with elevated metallothionein and cortisol levels in the fish brain after ZnCl_2_ exposure. Acetylcholinesterase (AChE), another essential cholinergic enzyme responsible for terminating neurotransmission by the cholinergic motor neurons, was significantly elevated after ZnCl_2_ exposure, altering neuromuscular activity and the behavioral response. In addition to these findings, the neurotoxic mechanism of the ZnCl_2_ exposure also modulates and induces oxidative stress in zebrafish models and causes a reduction in the antioxidant defense system (glutathione and superoxide dismutase) and elevated reactive oxygen species and malondialdehyde levels [12].

Numerous studies have been conducted to enhance the understanding of the behavioral manifestations induced by ZnCl_2_ exposure and have even correlated them with the pathology of AD. Sarasamma and collaborators tested the effects of zinc on zebrafish by exposing them to concentrations of 0.5, 1.0, and 1.5 mg/L zinc chloride for 24, 48, and 96 h, respectively [12]. Following exposure at all concentrations, there was a decrease in locomotor activity manifested by decreased swimming speed, increased latency time, and decreased total distance traveled. An anxiogenic effect of zinc was also observed. The mirror aggression test showed that the fish no longer exhibited the same normal aggressive behavior but instead exhibited a preference for spending increased time in the lower region of the tank. For memory testing, a negative stimulus was used. Following exposure, subjects no longer showed reluctance to enter the arm of the maze where the negative stimulus was applied, indicating that memory was impaired. Among these effects of zinc on zebrafish behavior is the reversal of activity correlated with normal circadian rhythm.

The current study aimed to further investigate the behavioral effects of zinc chloride on zebrafish, while also evaluating the potential impact of probiotics in this context. Recent literature offers evidence to support the beneficial response of these bacteria by relating gut health with the well-being of the nervous system [38].

The gut microbiota consists of a diverse array of bacterial species that colonize the gastrointestinal tract. It is important to understand that the gut microbiome (or gut microbiota) is composed of a varied community of bacterial species residing in the gastrointestinal tract that maintains a symbiotic association with the human host organism. The bulk of the microbiota belongs to the Firmicutes group (51%), which includes the species *Clostridium coccoides* and *Clostridium leptum*, as well as the genus Lactobacillus and the Bacteroidetes group (* 48%). The remaining 1% comprises less prevalent species, including those from the genera *Fusobacteria*, *Spirochaetes*, *Verrucomicrobia*, *Lentisphaerae*, and *Bifidobacteria*. Advances in modern sequencing technology have revealed that the gut microbiota comprises at least 1000 species and over 7000 strains, forming a population of 10^13^–10^14^ microorganisms. While there is significant inter-individual variability in gut microbial composition, the core functional roles remain conserved. This suggests the existence of a fundamental gut microbiota necessary to sustain essential physiological processes [39].

The gut microbiota can be viewed as a functional organ, contributing to essential physiological processes such as immune regulation, host metabolism, digestion, energy homeostasis, vitamin synthesis, and neurological development [40]. Despite the absence of gut microbiota analysis in this study, we acknowledge its critical role in understanding the effects of administered substances. Future research should address the diversity and composition of the microbiota, as this is essential for a more complete understanding of the observed effects. This research area warrants further attention in the specialized literature.

The multiple benefits of the bacteria used in our study have been highlighted in previous studies. For example, *Bifidobacterium animalis lactis* shows benefits at both the microbiological and immunological levels, being a possible very good adjuvant in the treatment of periodontitis and peri-implant mucositis [41], as well as showing potential in modulating the gut–brain–microbiota axis, increasing bowel movement, and decreasing intestinal transit time via the serotonin signaling pathway [19], suggesting that it may also be beneficial in accelerating the elimination of zinc chloride from the body. *Bifidobacterium longum*, another bacterium used in our study, can reduce depression scores in patients with irritable bowel syndrome (IBS), according to a 2017 clinical trial [42]. This reinforces our idea that this bacterium could have a beneficial effect on the toxicity induced by ZnCl_2_ exposure and on the specific AD-like behavior highlighted by Sarasamma [12]. Last but not least, the bacterium *Lactobacillus rhamnosus* shows benefits already described in the literature in many areas of interest, including having gastrointestinal, cardiovascular [43], and immunomodulatory effects [44]. This further strengthens the concept that it could have beneficial modulatory effects on the toxicity induced by zinc chloride exposure.

Moreover, a complex interplay exists between the gut microbiota and neurological health. Dysbiosis has been shown to influence the trajectory of neurological diseases, potentially even initiating pathological processes. Age-related reductions in gut microbial diversity are also implicated in the development of degenerative diseases [45]. Critically, evidence suggests a reciprocal relationship, with neurological insult and psychological status capable of modulating gut microbiota composition and potentially contributing to disease susceptibility [46].

Probiotics offer a promising avenue for mitigating heavy metal-induced neurotoxicity by modulating the gut microbiota. As demonstrated by Grochowska et al. [47], probiotic administration can significantly alter the gut flora composition, increasing beneficial bacteria like *Lactobacillus* and *Bifidobacterium*, while reducing the abundance of potentially harmful bacteria. This shift in microbial balance is crucial, as a disrupted gut microbiome can exacerbate heavy metal toxicity. Probiotics can enhance the gut barrier function, limiting the absorption of heavy metals into the bloodstream and reducing their access to the central nervous system [48]. Furthermore, the gut microbiota plays a key role in the production of neuroactive compounds, and probiotic-induced changes in gut flora can influence the levels of these metabolites, potentially counteracting the neurotoxic effects of heavy metals [49]. By restoring a healthy gut microbiome, probiotics can indirectly protect against heavy metal neurotoxicity through multiple interconnected pathways.

Analysis of behavioral parameters during the social preference test demonstrated no significant treatment-related effects on velocity, distance traversed, or inactivity duration. Regarding the number of entries, the administration of ZnCl_2_ at a 1.0 mg/L concentration appeared to have an anxiolytic effect, as opposed to the administration of probiotics alone, which made the subjects anxious. The time spent present in the left arm, however, suggests that the combination of ZnCl_2_ at a 1.0 mg/L concentration plus the probiotics had a cumulative anxiolytic effect on the fish that was stronger than the probiotics alone. The suspicion of an anxiogenic effect due to exposure to zinc chloride at a concentration of 1.0 mg/L is also supported by the significantly lower total distance traveled by that group than the control group in the long-term memory test aquarium, where again the administration of probiotics restored this parameter, thus reinforcing the notion that probiotics could have an anxiolytic effect but only in the case of exposure to ZnCl_2_ at 1.0 mg/L.

Probiotics alone did not influence sociability either, as the literature suggests [50]. However, administration of probiotics after intoxication with 1 mg/L ZnCl_2_ showed a positive trend in this type of behavior. In zebrafish, a six-week exposure to ZnCl_2_ at concentrations of 1.0 and 1.5 mg/L resulted in both anxiety-like behaviors and modified social preferences across both exposure groups according to a study [51].

In our study, the behavior of the subjects during the short memory test shows a decrease in velocity after the administration of the ZnCl_2_ in the 1.0 mg/L concentration, as well as after the administration of the probiotics too. The distance moved was also significantly reduced after the administration of both substances. Interestingly, the probiotics alone have had a similar effect regarding the shortening of the distance moved by the fish. According to the research articles studied, zinc chloride was shown to notably impact a wide range of behaviors, such as aggressivity, circadian rhythm disruption, predatory avoidance, motility, and sociability, even at the lowest of concentrations, while parameters such as memory and recognition are dose-dependent and therefore more visible at higher concentrations.

The control group’s differential exploration of the novel and familiar arms suggests the retention of information over the short term. The fish who were given 1.0 mg/L ZnCl_2_ spent the most time in the familiar arm, suggesting the loss of short-term memory. Following probiotic treatment, there was an observed increase in time spent in the new arm, which may suggest a potential influence on memory.

The long-term memory test was based on suggestions in the literature for this type of experiment [52], and the reason for opting for different shapes and colors of the objects was the documented ability of the zebrafish to distinguish between familiar and novel objects that present variations; essentially this species is capable of recognition memory [53,54,55]. Regarding the distance moved in the long-term memory test, it is evident that the group that received the higher concentration of ZnCl_2_ had its mobility negatively affected. After the probiotic treatment, a positive effect is observed, manifested as an increase in the distance moved. Long-term memory was improved in the subjects that received only probiotics, as well as in the group that received the ZnCl_2_ in a 1.0 mg/L concentration followed by the administration of probiotics. As the literature suggests, the difference in time spent exploring novel and familiar objects in zebrafish is evidence not only of the ability to remember but also to recognize objects. This suggests that familiar objects are recognized if the fish spend more time near the novel object, otherwise memory deficits occur [56].

The hypothesis behind this finding is that probiotic supplementation may have beneficial effects on zinc chloride poisoning. The protective potential of probiotics against metal toxicity is hypothesized to be mediated by their significant surface-binding capacity. This capacity is largely a function of the adhesive properties of S-layer proteins located within their cell walls. Both in vitro and in vivo investigations have shown that probiotic cultures can bind and/or degrade a range of toxic substances, resulting in reduced toxicity [57]. It is worth remembering that the gut microbiota–brain axis has a potentially important impact on the central nervous system through immunity. Studies have identified that the alpha diversity of the gut microbiota could even be a promising predictor for neurological spectrum diseases such as schizophrenia, Alzheimer’s, or multiple sclerosis [58]. The results show that short-term exposure to concentrations of 0.5 and 1 mg/L induces behavioral changes in zebrafish and that the administration of probiotics for seven days after intoxication shows a potential benefit in this regard. For example, regarding long-term memory, zinc chloride 0.5 mg/L had no effect on memory but probiotic supplementation improved it, while zinc chloride at 1 mg/L affected memory and probiotics had a beneficial effect to reverse the negative effects of heavy metals exposure.

## 5. Conclusions

The present study compared the effects of short-term exposure to zinc chloride and the effects post-administration probiotics on sociability and short- and long-term memory in zebrafish models. Regarding the social preference test, zinc chloride at a concentration of 1.0 mg/L caused a decrease in social behavior. Additionally, zinc exposure at 1.0 mg/L followed by probiotic administration resulted in the social behavior impairments from ZnCl_2_ exposure, also showing a possible anxiolytic effect that was more pronounced than in fish treated with probiotics alone.

In terms of memory performance, short-term memory was significantly impaired at both 0.5 mg/L and 1.0 mg/L ZnCl_2_. However, long-term memory was affected only at 1.0 mg/L, while no significant impairment was observed at 0.5 mg/L. No significant improvements in memory function were detected following probiotic treatment, and probiotics alone did not show notable beneficial effects on cognitive performance.

In summary, short-term exposure to zinc chloride induces behavioral changes in zebrafish, particularly affecting memory and social behavior. Although probiotic post-treatment showed some potential anxiolytic effects, the role of probiotics in reversing zinc-induced memory impairments remains uncertain. Further studies are needed to fully elucidate the mechanisms through which probiotics might modulate the effects of zinc toxicity.

## Data Availability

The data presented in this study are available on request from the corresponding author.
